# GR-2397: Review of the Novel Siderophore-like Antifungal Agent for the Treatment of Invasive Aspergillosis

**DOI:** 10.3390/jof8090909

**Published:** 2022-08-27

**Authors:** Karen Joy Shaw

**Affiliations:** Hearts Consulting Group, LLC, Poway, CA 92064, USA; kjshaw3@yahoo.com

**Keywords:** GR-2397, VL-2397, ASP2397, Sit1, siderophore, ferrichrome, antifungal, *Aspergillus*

## Abstract

GR-2397 (previously VL-2397, ASP2397) is a first-in-class antifungal agent for the treatment of invasive aspergillosis. This siderophore-like molecule resembles ferrichrome; however, it is differentiated by three amino acid changes and an aluminum rather than iron chelate. GR-2397 is transported into fungal cells via the Sit1 transporter, which is not found in humans, leading to fungal specificity. Although the precise mechanism of action is currently unknown, GR-2397 is active against *Aspergillus* spp. including azole-resistant strains, *Fusarium solani*, and *Candida glabrata* in addition to other organisms. Efficacy has been demonstrated in several animal models of invasive aspergillosis, including a 24 h delayed-treatment model where rapid fungicidal activity was observed. Phase 1 single- and multiple-ascending intravenous dose studies showed that GR-2397 was safe and well-tolerated in humans. No signs of GR-2397 accumulation were observed following IV infusions of 300, 600, and 1200 mg every 24 h (q24h) for 7 days. The favorable safety, tolerability and drug–drug interaction profile, along with good tissue distribution, support further development of GR-2397 as a new treatment option for patients with invasive aspergillosis. This systematic review summarizes the published findings of GR-2397.

## 1. Introduction

There is an urgent need for the development of new antifungal agents for the treatment of life-threatening invasive fungal infections (IFI), which are becoming more prevalent due to an increased use of immunosuppressive regimens in medical interventions. IFI are more common among patients with impaired immune function, including acute leukemia patients, solid-organ or hematopoietic stem cell transplant recipients, patients receiving cancer chemotherapy, or patients with other underlying diseases that lead to immunosuppression. Global estimates of life-threatening invasive fungal infections exceed 3 million infections per year and include 3 million cases of chronic pulmonary aspergillosis and >300,000 cases of serious and life-threatening invasive aspergillosis (IA) infections [1]. The associated mortality of IA has been reported to be 30–80% [2] or 30–95% [3] In a study of 983 proven/probable IFI identified in 875 hematopoietic stem cell transplant recipients, IA (43%), invasive candidiasis (28%), and zygomycosis (8%) were the most common [4]. Of the IA cases, *A. fumigatus* was the most common pathogen (44%), with *A. flavus* (7%), *A. niger* (9%), *A. terreus* (5%), other species (3%), multiple species (6%), and unidentified *Aspergillus* species (26%) comprising the rest of the 425 aspergillosis infections [4]. Similar trends were seen in a study of *Aspergillus*-positive cultures from 1209 patients: *A. fumigatus* (67%), *A. flavus* (16%), *A. niger* (5%), *A. terreus* (3%), *A. nidulans* (1%), other species (1%), and not identified (7%) [5].Current treatment options for IA include azoles (voriconazole, isavuconazole, itraconazole, posaconazole) and polyenes (amphotericin B deoxycholate or lipid formulations); however, despite treatment, mortality rates remain high (30–40%) [6]. A high percentage of fatalities (>50%) is still observed in invasive pulmonary aspergillosis (IPA) among severely immunosuppressed patients, such as neutropenic, leukemic and transplant patients [7]. In addition, these therapies are limited by renal and liver toxicity, drug–drug interactions, pharmacokinetic variability resulting in the need for drug monitoring, and emerging drug resistance [7]. For example, triazole-resistant *A. fumigatus* are a growing concern, primarily resulting from prolonged azole exposure in patients with chronic pulmonary aspergillosis and environmental exposure of isolates to triazoles used in agriculture [8].

Isavuconazole was the most recently approved treatment option for IA (2015); however, the pipeline remains sparse with few antifungals currently in clinical development [9].GR-2397 (previously ASP2397/Astellas and VL-2397/Vical) is the first of a new class of siderophore-like hexapeptide antifungal agents being developed for the treatment of serious fungal infections. GR-2397 is differentiated by its novel mechanism of action that confers rapid fungal cell killing, a low propensity for cytochrome P450 drug–drug interactions, and activity against difficult-to-treat resistant fungal pathogens. Phase 1 IV single-ascending dose and multiple-ascending clinical trials were successfully completed [10], and GR-2397 is currently in development by Gravitas Therapeutics, Inc. for the treatment of IA.

## 2. Literature Review

A PubMed search for information on GR-2397, VL-2397 and ASP2397 identified 14 citations containing information on structural evaluation, antifungal spectrum, resistance, animal models of infection, pharmacokinetics/pharmacodynamics and clinical trials. Evaluation of proceedings from conferences including Trends in Medical Mycology (TIMM), International Conference on Antimicrobial Agents and Chemotherapy (ICAAC), ASM Microbe, IDWeek, MSGERC and other international symposia identified 4 oral and 10 poster presentations of in vitro studies, efficacy studies, chemical properties, DMPK, ADME and clinical trials. Data from abstracts or presentations that were subsequently published were excluded from analysis, as were review articles.

## 3. Patent Filing and In-Licensing Arrangements

United States Patent 8,241,872 was obtained 14 August 2012 by Astellas Pharma, Inc. (Astellas, Tokyo, Japan) for the production of an antifungal agent (compound “B”) from strain *Acremonium persicinum* MF-347833 [11]. Earlier US and Japanese application dates suggest that the original discovery was prior to 2008. Of note is that MEC (minimum effective concentration) was used as the endpoint in the in vitro evaluation in the patent (0.31–0.39 µg/mL for species of *Candida* and 0.2–0.78 µg/mL for *Aspergillus* species) [11]; however, since this value was determined for both yeasts and molds, it is not clear whether the endpoint represents conspicuously aberrant growth of hyphae as measured for the echinocandins and manogepix against molds, 50% growth inhibition, or alternatively 100% inhibition as measured for the amphotericin B, itraconazole, posaconazole and voriconazole. The first posters were presented at ICAAC 2014 describing the in vitro and in vivo activity of ASP2397 [12,13]. In 2015, Vical Incorporated (Vical) in-licensed ASP2397 from Astellas, renaming it VL-2397. In 2016, Vical conducted Phase 1 clinical studies to evaluate the safety, tolerability and pharmacokinetics of intravenous VL-2397 administered once daily for up to 7 days. In October 2017, Vical announced that the U.S. Food and Drug Administration (FDA) had advised that VL-2397 would be eligible for a Limited Use Indication (LUI) approval assuming a successful outcome of a single Phase 2 trial carried out in accordance with a protocol and statistical analysis plan consistent with the Agency’s advice. In early 2018, Vical initiated a registrational Phase 2 clinical study evaluating VL-2397 as a potential first-line treatment for IA in immunocompromised adults with acute leukemia or recipients of an allogeneic hematopoietic cell transplant. In February 2019, Vical announced that it discontinued the Phase 2 clinical trial, a decision made to conserve cash resources following the unsuccessful completion of a Phase 3 trial for a separate program unrelated to GR-2397, as well as due to low patient accrual rates in the GR-2397 Phase 2 trial. In June 2019, Vical and Brickell Biotech, Inc. (Brickell) announced a merger agreement. Finally, in December 2021, Gravitas Therapeutics Inc. (Gravitas) announced that it had purchased the rights to VL-2397 from Brickell, renaming it GR-2397. Gravitas was founded specifically to acquire and rapidly return GR-2397 to the clinic, for the treatment of immunocompromised patients with serious fungal infections, including patients who are unable to receive one of the currently available antifungal agents due to resistance and/or intolerance.

## 4. Discovery of GR-2397 and the Activity of Related Analogs

GR-2397 is a non-ribosomally synthesized cyclic hexapeptide natural product (compound B) isolated from the Malaysian leaf litter fungus, *Acremonium persicinum* MF-347833 [14]. It was identified by researchers at Astellas who first screened culture broths of 310 geographically diverse fungal species for in vitro activity against *A. fumigatus* FP1305, and then identified active broths in an *A. fumigatus* silkworm larvae infection model [15]. Although attempts to purify the active fraction through in vitro antifungal screening failed, the silkworm model was successfully used to guide the purification of the therapeutically active GR-2397 [15,16].

**Figure 1:**
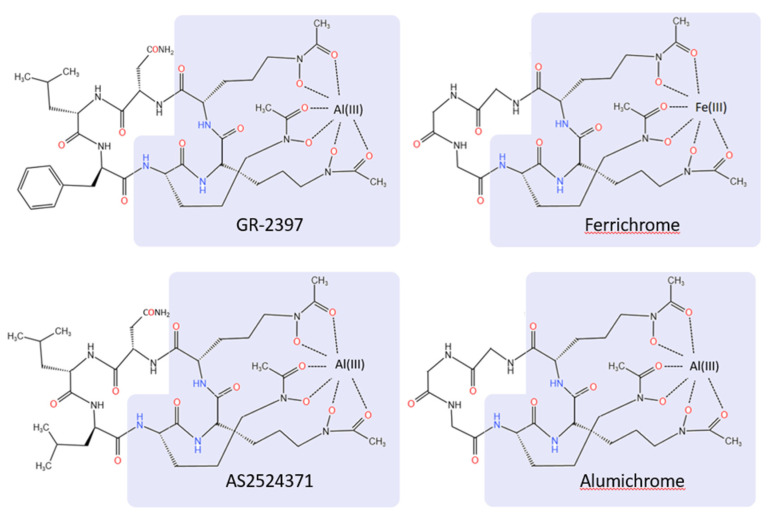
The structures of related ferrichrome-like molecules. The three Nε-acyl-Nε-hydroxy-ornithines responsible for metal chelation are shown in the highlighted background.

GR-2397 is structurally related to ferrichrome, a low-molecular-weight hydroxamate siderophore (Figure 1). Siderophores are produced by bacteria, fungi and plants in response to low iron levels, and are taken up by these organisms to scavenge these essential ions from the environment. The GR-2397 cyclic hexapeptide is composed of L-asparagine, L-leucine, D-phenylalanine and three Nε-acyl-Nε-hydroxy-ornithines (background highlighted), the latter of which chelate the aluminum ion [chemical name Cyclo{-Asn-Leu-d-Phe-[(N5-acetyl-N5-hydroxy-Orn)-(N5-acetyl-N5-hydroxy-Orn)-(N5-acetyl-N5-hydroxy-Orn)]-}*Al(III)] (Table 1) [14]. In contrast, ferrichrome is a cyclic hexapeptide composed of three L-glycine and three Nε-acyl-Nε-hydroxy-ornithines residues that utilize the same hydroxamic acid groups to bind Fe^3+^ (Figure 1) [17].

Different strains of *A. persicinum* have been shown to produce a number of metabolites related to ferrichrome [14,18,19,20]. These molecules share the three hydroxy ornithine residues that co-ordinate the metal ion but differ in the sequence of the other three amino acids. They have been referred to as acremonpeptides in some publications [18,19,20]. The apo form of the molecules generally lack or have poor antifungal activity. The first publication identified the structure of GR-2397 derived from *A. persicinum* MF-347833 [14]. Although the isolated GR-2397 active natural product was shown to be a siderophore-like molecule with an Al^3+^ chelate, the researchers speculated that *A. persicinum* produced the metal-free form of the hexapeptide, but addition of Al^3+^ ion to the culture medium increased the production of GR-2397 [14]. GR-2397 does not inhibit the growth of *A. persicinum,* suggesting the producing strain also encodes a resistance mechanism [20]. Its biological role in the producing strain has been hypothesized to be a defense metabolite [20].

Derivatives were also prepared that contained Fe^3+^ (AS2488053), Ga^3+^ (AS2529132), or lacked a chelating metal (AS2488059) (Table 1). A recent study showed that both the Al^3+^ chelate (GR-2397) and the Fe^3+^ chelate (AS2488053) were produced by *A. persicinum* MF-347833, and that incubation in iron-depleted media resulted in an 80-fold increase in the mRNA that encodes *SID1*, the genetic locus responsible for the production of these molecules [20]. Although these two metabolites (Fe^3+^ and Al^3+^ chelates) were largely secreted into the culture medium, a significant amount was still present in the mycelium. This contrasts with the related siderophore molecule, ferricrocin, produced by the same *A. persicinum* strain, where 10-fold higher mRNA levels derived from the corresponding biosynthetic *SID2* locus were induced when this organism was incubated under iron-replete conditions [20]. Ferricrocin was primarily detected in the mycelia, which the authors suggested served as intracellular/iron sequestering function for this molecule, whereas AS2488059 may primarily serve as an extracellular defensive metabolite and iron courier.

The inhibitory activities of GR-2397 and related molecules vs. *A. fumigatus* FP1305 are summarized in Table 1. Both the Al^3+^ and Ga^3+^ forms of the molecule showed antifungal activity against this strain when evaluated in RPMI media and RMPI + mouse serum (MS) [14]. The apo form of the molecule (AS2488059) was active in RPMI, but not active in the presence of mouse serum. Nakamura et al. [14] suggested that AS2488059 chelated free aluminum or ferrous ion in the RPMI culture medium, whereas there are not enough free metal ions in the presence of mouse serum, resulting in maintenance of the (inactive) apo form. The difference in MIC between the GR-2397 and AS2488053 (the Al^3+^ and Fe^3+^ forms of the molecule, respectively) suggest the role of Al^3+^ in activity. However, AS2524371, which contains a D-Phe to D-Leu substitution, and alumichrome (L-Gly, L-Gly, L-Gly) are also inactive (Table 1), suggesting that Al^3+^ is not solely responsible for the antifungal activity, but instead the composition of the variable three amino acid backbone, and specifically the D-Phe residue of the hexapeptide, plays an important role in antifungal activity. Similarly, the activities of acremonpeptides E and F, along with their apo, Al^3+^, Fe^3+^, and Ga^3+^ hydroxamate siderophore cyclopeptides, were evaluated against *A. fumigatus* [19]. These molecules are similar in structure to GR-2397 in that they all contain three Nε-acyl-Nε-hydroxy-ornithines (Figure 1) but differ in the sequence of the remaining amino acids (Table 1). Acremonpeptide E contains L-Ala, L-Leu, D-Phe, whereas acremonpeptide F contains L-Ser, L-Leu, D-Phe. Although activity was measured in non-standard media, the data in Table 1 shows that the Al^3+^ and Ga^3+^ chelates demonstrate antifungal activity (1 µm), the apo forms are 10-fold less active (10 µM), and the Fe^3+^ versions of these molecules have no antifungal activity (>30 µM), [19] (Table 1). Taken together, these data are consistent with the important role of the D-Phe residue in the activity of these molecules.

Acremonpeptides A, B, C, and D have been isolated that contain other amino acid substitutions: A) L-Phe, L-Leu, L-Ser; B) L-Phe, L-Leu, L-Ala; C) L-Phe, L-Leu, L-Phe; and D) L-Phe, L-Leu, L-Trp [18]. These molecules were reported to have no antibacterial or antifungal activity. However, since they were evaluated against two strains of *C. albicans* and not against *A. fumigatus*, and since GR-2397 is also inactive against *C. albicans*, it is difficult to draw any conclusions about the influence of these amino acid substitutions on activity vs. molds [18]. Interestingly, these molecules were reported to have low µM activity against Herpes Simplex Virus [18].

**Table 1:** Structure and activity of siderophore analogs.

Compound	Amino Acid Sequence ^1^ and Chelation Status						MIC (µg/mL) ^2^ *A. fumigatus* FP1305 ^2^ (+MS)	Reference
	Asn, Leu, D-Phe	Asn, Leu, D-Leu	Gly, Gly, Gly	Gly, Ser, Gly	Ala, Leu, D-Phe	Ser, Leu, D-Phe		
AS2488059	--						0.78 (>50)	[14]
AS2488053	Fe^3+^						>50 (25)	[14]
GR-2397	Al^3+^						0.78 (0.39)	[14]
AS2529132	Ga^3+^						0.78 (0.2)	[14]
AS2524371		Al^3+^					>50 (NT)	[14]
Ferrichrome			Fe^3+^				--	[20]
Alumichrome			Al^3+^				“inactive”	[14]
Deferriferrichrome			--				“inactive”	[14]
Ferricrocin				Fe^3+^			--	[20]
							MIC (µM) ^3^ *A. fumigatus* ATCC 204305	
Acremonpeptide E					--		10	[19]
Acremonpeptide E					Fe^3+^		>30	[19]
Acremonpeptide E					Al^3+^		1.0	[19]
Acremonpeptide E					Ga^3+^		1.0	[19]
Acremonpeptide F						--	10	[19]
Acremonpeptide F						Fe^3+^	>30	[19]
Acremonpeptide F						Al^3+^	1.0	[19]
Acremonpeptide F						Ga^3+^	1.0	[19]

^1^ L-amino acids unless otherwise indicated. All of the molecules contained the three Nε-acyl-Nε-hydroxy-ornithines, as shown in Figure 1. ^2^ CLSI M38-A3 [21] standard reference method (+mouse serum). ^3^ Media contained 10.4 g/L of RPMI-1640 medium, 6.7 g/L of Yeast Nitrogen Base, 1.8% (*w*/*v*) glucose, and 40 mM HEPES (pH = 7.1). The MIC was read at 24 h using resazurin at 0.002% (*w*/*v*) of final concentration [19].

## 5. Uptake of GR-2397 Is Mediated by the Sit1 Transporter

Although the mechanism of action has not been fully characterized to date, the mechanism of transport of GR-2397 has been identified in *A. fumigatus*. Since GR-2397 resembles ferrichrome, Dietl et al. isolated mutants of the siderophore transporters, Sit1 and Sit2 [22]. They found that an *A. fumigatus*Δ*sit1* mutant was resistant to GR-2397 (MIC >16 µg/mL), suggesting that the Δ*sit1* mutant fails to take up GR-2397 [22]. This is in contrast to the Δ*sit2* mutant, where the GR-2397 MIC was equivalent to wildtype strain (MIC = 1 µg/mL). Importantly, mammalian cells lack a siderophore transport mechanism such as Sit1, which results in an inability to transport GR-2397 by this mechanism, and thus target-based toxicity is predicted to be low. This is consistent with a lack of mammalian cell cytotoxicity at concentrations tested up to 50 μg/mL [16]. A description of the cell lines utilized for cytotoxicity assessment has not been published. Thus, it is difficult to assess whether this measurement is reflective of effects on normal human tissues.

Additional studies examined antifungal activity in a strain where *SIT1* expression was under control of the xylose-inducible *xylP* promoter [22]. In that strain, the activity of the Fe^3+^ analog AS2488053 was equally active as GR-2397 (Al^3+^). These data suggested that iron import affects AS2488053 susceptibility. Further, AS2488053 repressed expression of *SIT1* and other iron-repressible genes but increased the iron-inducible *CYCA* gene. The authors concluded that iron import mediated by AS2488053 uptake caused repression of *SIT1*, further blocking uptake of the drug. Thus, intracellular AS2488053 concentrations would be expected to be significantly lower than that of GR-2397, explaining the differences in antifungal activity between the Al^3+^ and Fe^3+^ forms of the molecule. 

The uptake and activity of GR-2397 was also evaluated against other species [12]. Whereas both uptake and antifungal activity were observed for *C. glabrata*, neither uptake nor antifungal activity were observed for *C. albicans* [12]. Microorganisms produce a variety of siderophore transporters that are differentially regulated, localized, and demonstrate different substrate specificities. Previous analysis showed that the specificity of a transporter for a particular substrate could not be predicted on the basis of protein sequence similarity [23]. Despite the fact that the *S. cerevisiae* genome encodes four siderophore transporters including two that transport ferrichrome (Arn1 and Sit1/Arn3), it is intrinsically resistant to GR-2397 [22,24]. When the Sit1 ortholog, derived from either *A. fumigatus* or *C. glabrata*, was cloned into *S. cerevisiae*, concentration-dependent growth inhibition was observed [22]. These data suggest that although the intracellular target of GR-2397 is present in *S. cerevisiae*, the wildtype strain is unable to take it up, resulting in resistance. 

*C. glabrata* and *C. albicans* each encode a single Sit1 protein; however, *C. glabrata* is susceptible to GR-2397 while *C. albicans* is resistant [22]. BLASTP analysis (https://blast.ncbi.nlm.nih.gov/Blast.cgi accessed on 17 August 2022) of the Sit1 orthologs *S. cerevisiae* Arn1 (NP_011823.1), *S. cerevisiae* Arn3/Sit1 (NP_011823), *C. albicans* (XP_716020.1), *C. glabrata* (XP_445865.1), *A. fumigatus* (XP_748904.1) and *A. flavus* (XP_041141397.1) demonstrates that the *S. cerevisiae* (resistant) and *C. glabrata* (susceptible) have greater sequence identity to each other than to the *C. albicans* (resistant) sequence (Table 2).

There are several hypotheses that could explain the phenotypic differences observed between susceptible and resistant species: (1) some fungal species may not express Sit1 orthologs under the conditions used to evaluate susceptibility, (2) the Sit1 ortholog may not recognize GR-2397 as a substrate for uptake, or (3) the intracellular target may differ within fungal cells such that GR-2397 interaction occurs in some species and not in others. Additional studies are necessary to differentiate these or other possibilities.

## 6. Mechanism of Action

In wild-type *A. fumigatus*, GR-2397 accumulates intracellularly and kills the fungal cell through an as yet unidentified mechanism of action. 

### 6.1. Intracellular Accumulation

Previous studies by Astellas demonstrated that GR-2397 accumulates intracellularly [12,25]. GR-2397 was added to *A. fumigatus* germinated conidia of a wild-type strain and a *sit1* transporter mutant and grown on a membrane filter. Over the course of 4 h, cultures were separated into medium and cell fraction and the concentrations of GR-2397 at 0, 1, 2, and 4 h were measured by LC/MS/MS. By 4 h, 63.33% of the GR-2397 was found in the cell fraction of the wildtype strain, whereas 4.735% was observed in the *sit1* transporter mutant [25]. Similarly, AS2524371 (the Al^3+^ chelate which contains one amino acid substitution) was also taken up by the wildtype strain (36.77%) [25]. 

Moore et al. showed that in *S. cerevisiae*, both ferrichrome [Fe^3+^] and alumichrome [Al^3+^] (Figure 1) accumulate in the cytosolic fraction as the chelated molecules, with almost no accumulation in the vesicular fraction [26]. These chelates (>85%) were maintained even after 4 h incubation in iron-poor media, likely serving as an iron-storage mechanism. They also showed that the cytosolic alumichrome did not undergo ligand exchange with cytoplasmic pools of free ferric iron [26]. 

**Figure 2:**
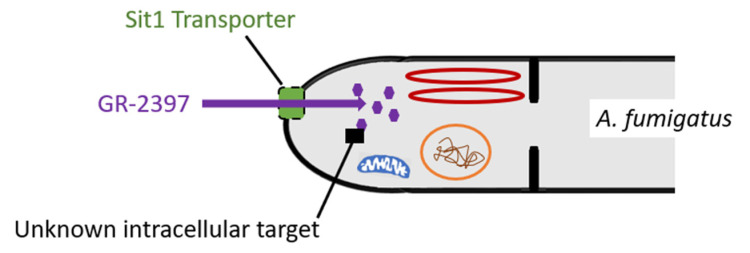
Transport and mechanism of action of GR-2397.

Despite the fact that both GR-2397 [Al^3+^] or AS2488053 [Fe^3+^] accumulates intracellularly, only GR-2397 demonstrates antifungal activity against *A. fumigatus*. Because analogs of GR-2397 such as AS2524371 (Table 1) have been identified that are taken up but do not exhibit antifungal activity, a putative intracellular target has been hypothesized (Figure 2) [12]. If GR-2397 accumulates in the cytosolic fraction similar to ferrichrome and alumichrome, it would suggest that its antifungal activity is due to interaction with a cytosolic component.

This target could potentially be identified using one or more techniques including: (1) isolation of additional mutants that lead to increased resistance to GR-2397, followed by whole genome sequencing to identify non-Sit1 mutants; (2) screening libraries of known *A. fumigatus* or *C. glabrata* mutants to identify those with increased (or decreased) susceptibility to GR-2397; (3) using labelled/tagged GR-2397 to identify proteins that bind to this molecule; and (4) identifying differences in metabolites or mRNA transcripts of cells grown in sub-MIC levels vs. no drug.

### 6.2. Rapid Inhibition of Hyphal Elongation

Hyphal formation is an important attribute of fungal infections, allowing invasion into host tissues. *C. albicans* mutants that are unable to form hyphae are often avirulent in systemic infection models [27]. During mold infections such as *A. fumigatus*, airborne conidia are inhaled and can germinate in patients with insufficient pulmonary defenses, such as those found in neutropenic patients. The subsequent formation and extension of hyphal can lead to further dissemination and systemic infections. Thus, antifungal agents that inhibit hyphal growth may reduce the establishment and progression of fungal diseases. 

To evaluate the effects on hyphal elongation, GR-2397 or voriconazole were added to *A. fumigatus*, *A. terreus* and *A. flavus* germinated conidia to a final concentration of 4× or 16× MIC and cultures were grown in human serum [25]. The area of growth was quantified (*n* = 4) by live cell imaging at 0, 2, and 8 h. Whereas addition of voriconazole resulted in some hyphal elongation at 2 h vs. no drug control, addition of GR-2397 rapidly blocked elongation and no increase in the T = 0 fungal area was observed either at 2 or 8 h [25]. The data suggest that GR-2397 has a faster onset and more effectively inhibits hyphal elongation of all three *Aspergillus* spp. than voriconazole, which may in turn translate into less penetration into host tissues. If so, these findings would have important implications for the treatment of IA.

### 6.3. Time-Kill Assay

Germinated *A. fumigatus* conidia were inoculated into human serum in 96 well microtiter plates, and increasing doses of GR-2397, voriconazole, posaconazole and amphotericin B (1/4× MIC, 1× MIC, 4× MIC and 16× MIC) were added [25]. Viable counts were measured at 0, 1, 2, 4, 6 and 8 h. GR-2397 demonstrated a dose-dependent rapid onset of fungal killing (e.g., reduced *A. fumigatus* by nearly 2 log_10_ CFU/mL within 8 h at 4× MIC), whereas the posaconazole required 16× MIC to achieve a 2 log_10_ CFU/mL reduction. The onset of killing was more rapid for GR-2397 than posaconazole. Amphotericin B (4× MIC, the highest concentration tested) and voriconazole (16× MIC) did not achieve a reduction of 2 log_10_ CFU/mL by 8 h. Similar results were obtained for GR-2397 against *A. terreus* [25].

## 7. Resistance

The potential for development of resistance to GR-2397 has not been fully investigated. Attempts to isolate *A. fumigatus* mutants with decreased susceptibility through serial passage (19 passages) on sub-inhibitory concentrations of GR-2397 did not alter susceptibility to the drug [25]. A Δ*sit1* transporter mutant, isolated after UV mutagenesis, demonstrated GR-2397 MIC values >16 μg/mL in RPMI media, whereas the susceptibility of the mutant to voriconazole was unchanged (0.25 μg/mL) [24]. This is consistent with a lack of cross-resistance since voriconazole does not use the siderophore mechanism of transport. A mutant constructed with a deletion of another siderophore transporter (Δ*sit2*) did not affect the GR-2397 MIC. Additional studies are necessary in key *Aspergillus* species to establish the frequency of GR-2397 resistance.

The identification of SIT1 as a mechanism of GR-2397 resistance leads to the question of whether or not these mutants may arise in patients and impact treatment outcomes. The growth phenotype of the *A. fumigatus* Δ*sit1* mutant was evaluated in complex media as well as several minimal media including high iron, iron sufficiency and iron starvation, and no differences between mutant and wildtype were observed [22].

Park et al. evaluated the virulence of *A. fumigatus sit1* and *sit2* deletion strains [28]. Although they reported that *sit1* and *sit2* mutants did not impact the mouse 10-day survival rate in an immunocompromised intranasal infection model, this failure to reach statistical significance may have resulted from the use of small numbers of animals (reported as *n* = 5 mice, although the data suggests additional mice may have been present in some of the cohorts) [28]. Whereas none of the mice infected with the wildtype strain survived past day 5, ~30% of the *sit1*Δ and *sit1*Δ/*sit2*Δ mice survived to day 10, suggesting that virulence may have been impacted. Median time to death was not reported. This study also evaluated the effects of conidial killing by neutrophil-like or macrophage-like HL-60 cells [28]. In this assay, statistically significant increases in killing were observed for the *sit1*Δ, *sit2*Δ and *sit1*Δ/*sit2*Δ mutants vs. wildtype. These data suggest that *A. fumigatus sit1* mutants arising in vivo may be less virulent.

Similarly, a *C. albicans* *sit1*/*sit1* deletion strain was constructed and virulence was assessed in an in vitro model in which a keratinocyte cell line was allowed to differentiate on an inert supporting membrane, resulting in a multilayered keratinocyte tissue [29]. While the wildtype strain *SIT1+* strain entered the upper cellular layers eventually causing extensive damage, the *sit1* mutant strain caused minimal damage and few *C. albicans* cells were observed in the upper layer of keratinocytes, even at later time points. However, in a mouse systemic infection model of candidiasis, no differences were observed in the virulence of the wildtype strain vs. the *sit1*/*sit1* deletion strain [29]. The authors suggest that a *sit1 Candida* mutant may impact epithelial invasion and penetration, whereas in blood or other organs where other sources of iron may be used, there may be less of an impact [29].

Deletion of the single *SIT1* locus of *C. glabrata* resulted in a strain that was unable to grow or use a xenosiderophore-Fe in iron deficient media [30]. Thus, it is unlikely that a *sit1* mutant would be viable under in vivo conditions where very low iron levels are present. In addition, the study also showed that SIT1 protein mediated ferrichrome utilization provided a survival advantage to phagocytosed *C. glabrata* cells [30].

Taken together, the data suggest that the SIT1 protein may provide a selective advantage during an infection. Additional studies are necessary to assess whether or not *sit1* mutants will arise clinically in different species and result in a potential resistance issue. The issue of resistance may be mitigated by the fact that GR-2397 demonstrates a rapid onset of fungal cell killing, which should rapidly lower the fungal burden. 

## 8. Spectrum of Activity

### 8.1. Effect of Different Media Conditions on GR-2397 Susceptibility Testing

The Nakamura et al. patent filing describing the cyclic peptide produced by *A. persicinum* utilized a MEC endpoint value in the assessment of antifungal activity of single strains of 5 yeast and 11 mold species [11]. The reported MEC values were 0.31–0.39 µg/mL for species of *Candida*, 0.2 µg/mL for *C. neoformans*, and 0.2–0.78 µg/mL for *Aspergillus* species. Activity was also observed for *Fusarium solani* (0.2 µg/mL) but not for *Rhizopus oryzae* (25 µg/mL). However, it is possible that the MEC endpoint represented 50% or 100% inhibition rather than the first microtiter well with aberrant growth of hyphae as measured for the echinocandins and manogepix, since both yeasts and molds were tested. Given that administration of GR-2397 results in rapid inhibition of fungal elongation [25], it may be worthwhile to evaluate the CLSI defined MEC endpoint for this new class of drugs.

The GR-2397 spectrum of activity has also been examined using standard CLSI methods [21], in heat inactivated human serum containing 20 mmol/L HEPES (pH 7.4) under 5% CO_2_ at 37 °C, and in iron-depleted media [25], where MIC endpoints were read at 100% inhibition. The human serum media were utilized based on the finding that *C. albicans* ATCC 90028 MIC values in this medium better correlated with the preclinical in vivo effects of fluconazole, itraconazole, ketoconazole, and amphotericin B [31]. However, the relevance of this finding to the antifungal activity of GR-2397 against *Aspergillus* and other molds needs to be further assessed. MIC values (100% inhibition endpoint) against single strains ranged from 1–2 µg/mL for *Aspergillus* species, 2 µg/mL for *F. solani*, *Cryptococcus neoformans* and *Trichosporon asahii*, 1 µg/mL for *Candida kefyr* and *C. glabrata*, and >16 ug/mL for other *Candida* species. These investigators also evaluated activity against larger panels of *Aspergillus* and compared heat-inactivated human serum to standard CLSI reference methods [21]. MIC_90_ values in human serum and by CLSI standard methodology, were, respectively: *A. fumigatus* (*n* = 49) 2, 0.5 µg/mL; *A. niger* (*n* = 20) >16, >16 µg/mL, *A. flavus* (*n* = 17) 2, 16 µg/mL; *A. terreus* (*n* = 20) 4, 1 µg/mL. The comparator drugs voriconazole, itraconazole, posaconazole and amphotericin B showed lower (or equal) MIC values using RPMI medium vs. human serum medium, presumably due to effects of protein binding. However, although some trends were observed in the directionality of changes in GR-2397 MIC values determined in the two media, they were not consistent, and some puzzling inconsistencies were observed with some species. These may be related to differences in compound uptake, target, degradation, efflux, or in regulatory responses in different species.

Another study examined antifungal activity using non-standard methods (50% suppression of growth in the defined media Czapek-cox (CD) or M media, and also examined the effects of low iron conditions [20]. Although only limited studies using iron deficient or iron supplemented media were conducted, the data suggest that assessment of antifungal activity in low iron conditions results in greater susceptibility, which would be expected for a siderophore-like molecule dependent on the Sit1 protein for uptake. For example, comparing assessments in RPMI vs. RPMI + apo-transferrin media, MIC values decreased for *A. fumigatus* (4-fold), *A. flavus* (>4-fold), *A. terreus* (8-fold), *A. niger* (>2-fold) and *A. nidulans* (>4-fold) [25]. However, CLSI-defined MEC values (the lowest drug concentration at which short, stubby, highly branched hyphae are observed) were never evaluated in media containing different iron levels, and the impact on this potential endpoint cannot currently be assessed. Despite being a Phase 2 ready agent, the most appropriate methodology for antifungal susceptibility assessment has not been finalized, nor have QC strains been assayed or multi-laboratory studies been published. Thus, it is difficult to fully assess the in vitro spectrum of activity of this new class of agents.

### 8.2. Activity against Azole- and Amphotericin B-Resistant Aspergillus spp.

GR-2397 was evaluated against a panel of well-characterized *Aspergillus*, including azole resistant strains [32]. Both EUCAST EDEF9.2 [33] and CLSI M38-A3 [21] reference methods were utilized along with modified EUCAST methods where either a 10-fold-lower inoculum or a spectrophotometer 50% growth inhibition endpoint were evaluated. For *A. fumigatus*, the MIC_50_ value of 0.25 µg/mL (+/− 2-fold) was observed for 4 wild-type strains and 24 CYP51A azole-resistant mutant isolates across the 4 media. MIC values for *A. terreus* were somewhat higher than those for *A. fumigatus*, consistent with what had been previously observed [24]. MIC values for two wild-type *A. terreus* isolates ranged from 0.5–1 µg/mL using both CLSI and EUCAST methodologies, 0.5–4 µg/mL (lower inoculum EUCAST) and 0.25–0.5 µg/mL (EUCAST spec-50%). For a single *CYP51A A. terreus* mutant (M217I), MIC values were 2, >4, 4 and 4 using CLSI, EUCAST, EUCAST low inoculum and EUCAST spec-50% methodologies, respectively [32]. The data suggests the presence of an uncharacterized mutation in this strain since CYP51A is not seen as a target for GR-2397. However, another *A. fumigatus* isolate harboring a *CYP51A* (G54E) alteration was found to be resistant to GR-2397 (MIC > 4 µg/mL) by all methods. Thus, GR-2397 displayed in vitro activity against both *A. fumigatus* and *A. terreus* isolates and the activity was largely independent of the presence or absence of azole target gene resistance mutations.

## 9. In Vivo Efficacy

The efficacy of GR-2397 was examined in early treatment and delayed treatment models of IPA where mice were immunocompromised with cyclophosphamide and fungal conidiospores introduced via an intratracheal route (Table 3) [25]. GR-2397 (subcutaneous) and posaconazole (oral) treatments both demonstrated similar efficacy (100% survival) in an early treatment (6 h post-infection) IPA model.

GR-2397 (subcutaneous administration) also demonstrated significant efficacy in 24 h delayed treatment IPA models of infection using both *A. fumigatus* azole-refractory strain (20030) and azole-resistant (*CYP51A* mutant 25001) strains as the inoculum (Table 3) [25]. At day 10 in both models, 100% survival was observed when mice were treated with GR-2397 at 4 mg/kg or 8 mg/kg twice daily, whereas only 40% of the mice infected with the azole-refractory strain and no mice infected with the azole-resistant strain survived on posaconazole (10 mg/kg twice daily). A dose of 10 mg/kg/day posaconazole has been previously used to benchmark the orotomides since it achieves a clinically relevant exposure in mice [34] and results in suppression of galactomannan in murine models of IPA [35]. Analysis of lung fungal burden on day 3 of the delayed IPA treatment (azole-refractory) model showed that the twice daily 2, 4, and 8 mg/kg doses of GR-2397 resulted in a significant reduction in colony-forming units (CFU)/g lung versus the untreated control, with the higher two doses both resulting in >1 log_10_ CFU reduction [25].

In the azole refractory delayed treatment model, the combination of GR-2397 (4 mg/kg BID) plus posaconazole (10 mg/kg BID) or GR-2397 alone resulted in significantly improved survival (90%) vs. the untreated control (10%) and posaconazole alone (20% survival). Although these data are consistent with the lack of antagonism for these two drugs, they do not support the use of the two drugs in combination [25]. 

GR-2397 was also evaluated in a disseminated model of invasive candidiasis caused by both wildtype and multi-drug resistant *C. glabrata* (Table 3) [36]. Efficacy was assessed by evaluating kidney fungal burden on day 8 after twice daily doses of 2 mg/kg, 4 mg/kg, and 8 mg/kg doses of GR-2397, 20 mg/kg fluconazole, or 1 mg/kg caspofungin. Against the wild-type strain, a significant reduction of CFU/g of tissue was observed for all GR-2397 dosing groups (range mean 3.62–4.13 log_10_ CFU/g) vs. control (5.24 log_10_ CFU/g). Caspofungin also resulted in a significant reduction in CFU/g of tissue (3.67 log_10_ CFU/g) vs. control. However, fluconazole showed no significant efficacy in this model (4.85 log_10_ CFU/g). Against the caspofungin-/fluconazole-resistant MDR isolate, significant reductions in CFUs were also observed with all three doses of GR-2397 (4.30–5.14 log_10_ CFU/g) compared to control (6.63 log_10_ CFU/g), but not for caspofungin treatment (5.76 log_10_ CFU/g). Treatment with fluconazole did result in significant efficacy (5.34 log_10_ CFU/g) vs. control, despite a fluconazole MIC of 64 µg/mL against the infecting strain [30]. The results suggest that GR-2397 has the potential to be used for the treatment of invasive *C. glabrata* infections.

**Table 3:** Summary of animal models demonstrating GR-2397 efficacy.

Pathogen	Infection Type	Tx initiation PI ^1^	Efficacy Endpoint	Species	Reference
*A. fumigatus* (azole-susceptible)	pulmonary	6 h	survival	mouse	[25]
*A. fumigatus* 20030 (azole-refractory)	pulmonary	delayed treatment 1 day	survival, CFU lung	mouse	[25]
*A. fumigatus* 20030 (azole-refractory)	pulmonary	delayed +posaconazole combination treatment 1 day	survival	mouse	[25]
*A. fumigatus* 25001 (azole-resistant *CYP51A*)	pulmonary	delayed treatment 1 day	survival, CFU lung	mouse	[25]
*A. fumigatus*	disseminated	1 h	survival	silkworm larvae	[15]
*A. fumigatus*	disseminated	1 h	survival	mouse	[15]
*C. glabrata*	disseminated	1 day	CFU kidney and spleen	mouse	[36]

^1^ Time of treatment initiation post-infection. Subcutaneous dosing of GR-2397 was utilized in all of the models.

## 10. Determination of the Pharmacokinetic Driver of Efficacy

A multiple-dose study [37] of GR-2397 for the treatment of mice in the IPA model of *A fumigatus* FP1305 (MIC = 1 μg/mL in human serum [38]; MIC = 0.125 μg/mL CLSI method), was used to determine the PK/PD parameter that best predicted efficacy [37]. In this study, mice were immunocompromised with cyclophosphamide (200 mg/kg IP days −4 and +1) and infected intratracheally on day 0 with *A. fumigatus* FP1305. GR-2397 was administered subcutaneously on days 1, 2, and 3 post-infection. Daily dose sizes included 1, 2, 4, 8, and 16 mg/kg/day, and doses were fractionated daily, twice daily, and four times daily (a total of 15 doses tested). The lung fungal burden (LFB) was determined after sacrificing animals at day 4 (after 3 days of dosing) and evaluating the CFU/g tissue. LFB decreased in a dose-dependent manner, independent of dose frequency. When survival was examined in the same model (infection day 0, treatment days 1, 2, 3, evaluation of day 10 survival), survival was 0–30% (2 mg/kg/day), 30–70% (4 mg/kg/day) 90–100% (8 mg/kg/day) and 100% (16 mg/kg/day) [37].

Non-linear PK was observed due to concentration-dependent saturable plasma protein binding [37]. However, after adjusting for saturable protein binding, unbound PK profiles were linear and well-fitted to a 1-compartment model. The reduction in fungal burden correlated with the total daily dose of GR-2397. The best fit curve among PK parameters and antifungal lung burden (log CFU/g) was found for unbound AUC (r^2^ = 0.8978), whereas unbound C_max_ (r^2^ = 0.6904) and T > MIC (r^2^ = 0.4512) were less predictive.

## 11. In Vitro Safety Pharmacology

The potential for GR-2397 off-target activities was assessed in 54 radioligand-binding assays using a variety of receptors, ion channels, and transporters, as well as inhibitory activity in acetylcholinesterase and monoamineoxidase A and B enzyme assays [39]. At 30 µg/mL, no inhibition of greater than 30% was seen in any of the radioligand binding assays, nor was inhibition observed in the three enzyme assays. Therefore, the potential for off-target activity at therapeutic exposure levels is very low.

The inhibitory effects of GR-2397 on cytochrome P450 (CYP) metabolism were also evaluated [39]. CYP isozyme-specific probes were incubated with human liver microsomes and probe substrate metabolism was evaluated for: CYP1A2 (phenacetin), CYP2B6 (bupropion), CYP2C8 (paclitaxel), CYP2C9 (diclofenac), CYP2C19 ((S)-mephenytoin), CYP2D6 (dextromethorphan) and CYP3A (midazolam and testosterone) [39]. The IC_50_ values after pre-incubation with human liver microsomes (0 and 30 min) was >500 µM for all CYP enzymes including CYP3A (testosterone), with the exception of CYP3A (midazolam), which was 206 µM and 160 µM (after 0- and 30-min incubation, respectively). These data suggest that the propensity for drug-drug interactions by GR-2397 is low and thus treatment with GR-2397 may provide an advantage over azole therapies.

## 12. Phase 1 Clinical Trials

The Phase 1 clinical trial VL2397-101 was conducted by Vical (ClinicalTrials.gov NCT02956499). This first-in-human, randomized, double-blind, placebo-controlled dose-escalation study, was conducted in healthy adults to assess the safety, tolerability, and pharmacokinetics of single- and multiple-ascending intravenous doses [10]. A total of 96 subjects were enrolled in seven single-ascending dose (SAD) and four multiple-ascending dose (MAD) cohorts. Subjects were randomized in a 3:1 ratio to receive IV infusions of GR-2397 or placebo. Since the drug concentration was kept constant at 0.12 mg/mL (low dose cohorts 1–3) or 1.2 mg/mL (cohorts receiving ≥100 mg dose), infusion times varied from 6–240 min and the total volume administered ranged between 23–1000 mL [10]. SAD cohorts 1–7 received 3 to 1200 mg as a single dose, whereas MAD cohorts 8–10 received once daily doses of either 300, 600 and 1200 mg for 7 days. For MAD cohort 11, subjects received 300 mg T.I.D daily (total 900 mg) for 7 days followed by 600 mg once daily for an additional 21 days (total 28 days). 

Pharmacokinetic analysis showed that AUC_0-24_ and C_max_ values rose less than proportionally with increasing doses (3 to 1200 mg) and AUC_0-24_ and C_max_ values for the 7-day MAD cohorts were comparable to those observed for the corresponding SAD cohorts. AUC_0-24_ values were also less than dose-proportional for the 300, 600 and 1200 mg MAD doses, as was the rise in C_max_. A low variability in exposures was observed among subjects within a cohort or those given the same daily dose in the SAD and MAD studies. There was no apparent accumulation following multiple doses. Nonclinical plasma protein binding studies had previously showed that the major serum binding protein zinc-α2-glycoprotein (ZAG) bound GR-2397 at a very high affinity, and binding was saturated above the 30-mg dose [10]. This was confirmed in this Phase 1 study, where protein binding of GR-2397 was saturated between the 30-mg and 100-mg doses [10]. In a study of population PK, this protein binding was suggested to be the primary source of the nonlinearity [40]. 

Overall, GR-2397 dosing appeared to be safe and well tolerated in the healthy male and female subjects in this study. No serious adverse events (SAEs) occurred, and the majority of treatment related adverse events (TEAEs) were mild and self-limited, with the most common TEAE being infusion site reactions. Two of the subjects receiving the highest dose tested (1200 mg, MAD cohort) discontinued dosing due to TEAEs including: (a) elevation in serum creatinine from normal after the second dose of GR-2397, which returned to normal by day 17 without intervention; and (b) a severe generalized rash approximately 10 h after receiving the second dose of GR-2397. No additional subjects in any of the SAD or MAD cohorts who received GR-2397 experienced any laboratory- or vital sign-related TEAE related to the study drug. The results from these studies combined with the lack of CYP inhibition suggest that weight-based dosing, therapeutic drug monitoring, and dose adjustments will not be required [10,39].

## 13. Population PK Modeling, Probability of Target Attainment and Dose Selection for Phase 2 Clinical Trials

A total of 1908 plasma concentrations were collected from sixty-six healthy subjects from the 11 Phase 1 study cohorts (SAD and MAD). Population PK modeling showed that a nonlinear saturable binding model with 3 compartments fit the data well [40]. PK analysis showed that for the 300, 600 and 1200 mg SAD cohort, a mean AUC_inf_ (SD) of 104.3 (12.8), 150.6 (17.7) and 236.0 (46.7) mg·h/L were observed, respectively. On day 1, GR-2397 concentrations decreased to approximately 1 µg/mL by hour 16, regardless of QD dose level, with slow clearance over time, suggesting a rapid and saturable distribution phase. However, administration of 300 mg every 8 h achieved concentrations above 1 µg/mL over the entire 24-h period [40].

To support Phase 2 dose selection, data from the dose fractionation study in an *A. fumigatus* FP1305 mouse model of IA, which measured reduction in fungal lung burden on day 4 [37], and the Phase 1 study in healthy adult volunteers [10] were used to assess the probability of target attainment for 3 different dosing regimens (300, 600, and 900 mg QD) [38]. A two-compartment model with linear elimination and concentration-dependent binding in both central and peripheral compartments provided a robust fit to the data. The results of the dose fractionation study in mice indicated that fAUC_0-24_/MIC was the most predictive driver of efficacy (r^2^ = 0.708). In this model, a fAUC_0-24_/MIC ratio of 8.40 on day 1 was associated with a 2-log reduction in fungal lung burden on day 4 [37]. Target attainment simulations showed that once daily IV dosing of 600 mg would provide a robust (99.9%) target attainment (as measured by 2-log reduction in fungal burden) up to and including an *A. fumigatus* MIC of 4 μg/mL [38].

## 14. Phase 2 Clinical Trials 

ClinicalTrials.gov lists a single clinical trial (NCT03327727): A Phase 2 Study of VL-2397 Compared to Standard First-Line Treatment for Invasive Aspergillosis in Adults with Acute Myelogenous Leukemia, Acute Lymphocytic Leukemia, or Allogeneic Hematopoietic Cell Transplant Recipients. In this trial 600 mg GR-2397 was administered by IV infusion every day for 28 days (4 weeks), followed by 2 weeks of standard treatment (voriconazole, isavuconazole, or liposomal-amphotericin B). Active comparator consisted of standard treatments of voriconazole, isavuconazole, or liposomal amphotericin B administered every day for 42 days (6 weeks). The primary endpoint was all-cause mortality (ACM) at 4 weeks, with a key secondary endpoint of ACM at 6 weeks. The trial was terminated by Vical in early 2019 for business reasons. A reverse merger of Vical and Brickell was announced in June 2019 (https://brickellbio.com/vical-and-brickell-announce-merger-agreement accessed on 17 August 2022) with a focus on autoimmune and inflammatory disorders. Brickell sold VL-2397 to Gravitas in 2021 for further development (https://www.einnews.com/pr_news/557667225/gravitas-therapeutics-announces-purchase-of-first-in-class-antifungal-intended-to-treat-invasive-aspergillus-infections accessed on 17 August 2022).

## 15. Regulatory Status 

The U.S. Food and Drug Administration has granted Qualified Infectious Disease Product (QIDP), Orphan Drug and Fast Track designations to GR-2397 for the treatment of IA, and the IND remains open with the FDA (https://www.einnews.com/pr_news/557667225/gravitas-therapeutics-announces-purchase-of-first-in-class-antifungal-intended-to-treat-invasive-aspergillus-infections accessed on 17 August 2022). Prior to initiating the Phase 2 clinical trial, the FDA advised Vical that GR 2397 would be eligible for accelerated development via the Limited Population Pathway for Antibacterial and Antifungal Drugs (LPAD) with the objective of receiving registration for a Limited Use Indication (LUI). The LUI is a provision of the Limited Population Pathway established under the 21st Century Cures Act of 2016. Receiving the LUI would require a successful outcome of a single Phase 2 trial carried out in accordance with a protocol and statistical analysis plan consistent with the FDA’s advice and subject to a final determination by the FDA as to whether the drug is approvable after reviewing all relevant data. In the case of GR 2397, the limited population approval would be for treatment of IA in patients who are unable to receive treatment with one of the currently approved antifungal agents.

## 16. Discussion 

GR-2397 is a novel, first-in-class antifungal agent in clinical development to address IA infections, a disease with high mortality [2,3]. Although GR-2397 demonstrates in vitro activity against a wide range of clinically important yeast and molds, including *Aspergillus* spp., *C. glabrata*, and *F. solani*, assessment of antifungal activity remains challenging since tier 2 (multi-laboratory) testing has not been completed, nor have QC strains been defined for this new agent. Thus, the spectrum of activity is not fully understood for this siderophore-like agent whose activity against some species is dependent on iron levels in the media. Despite this limitation, efficacy has been observed in several animal models, including multi-drug resistant and other difficult-to-treat infections such as azole-resistant *A. fumigatus* and *C. glabrata*. Both improved survival, and reductions in kidney and lung fungal burdens have been assessed. It would be worthwhile to evaluate the efficacy of GR-2397 in additional animal models using other yeasts and mold pathogens to better understand the potential spectrum of this new agent.

The reduction in tissue fungal burden is remarkably rapid; thus, this rapid in vivo fungicidal activity should be considered a significant attribute of this drug if it translates to rapid fungal clearance in human infections. The lack of target-based cross-resistance to the other main classes of antifungal agents is an additional attribute, especially for organisms such as *A. fumigatus* and *A. terreus* where azole resistance may limit treatment options. 

An IV formulation of GR-2397 has been used in early development. In vitro acid stability experiments have not been performed, nor have PK or oral efficacy been evaluated. Thus, the potential for an oral formulation is unknown. Once-daily dosing in humans, a favorable drug–drug interaction profile with no significant CYP3A4 inhibition, a good nonclinical and Phase 1 safety profile together suggest that GR-2397 may be an important alternative to the treatment of *Aspergillus* fungal infections, and possibly other invasive infections as well, especially for the treatment of patients who cannot tolerate other marketed antifungal agents.

## Figures and Tables

**Table 2:** Percent sequence identity of siderophore orthologs.

	*S. cerevisiae* Arn1	*S. cerevisiae* Sit1/Arn3	*C. glabrata* Sit1/Arn3	*C. albicans* Sit1/Arn1	*A. fumigatus* Sit1	*A. flavus* Sit1
*S. cerevisiae* Arn1	100					
*S. cerevisiae* Sit1/Arn3	48.08	100				
*C. glabrata* Sit1/Arn3	72.45	49.73	100			
*C. albicans* Sit1/Arn1	51.19	47.17	48.94	100		
*A. fumigatus* Sit1	41.04	46.06	40.47	43.22	100	
*A. flavus* Sit1	38.80	45.24	39.89	40.13	66.67	100
